# A New Tool to Measure Malevolent Creativity: The Malevolent Creativity Behavior Scale

**DOI:** 10.3389/fpsyg.2016.00682

**Published:** 2016-05-18

**Authors:** Ning Hao, Mengying Tang, Jing Yang, Qifei Wang, Mark A. Runco

**Affiliations:** ^1^School of Psychology and Cognitive Science, East China Normal UniversityShanghai, China; ^2^American Institute for Behavioral Research and Technology, San DiegoCA, USA

**Keywords:** creativity, malevolent creativity, aggression, openness, extraversion

## Abstract

The present study developed the malevolent creativity behavior scale (MCBS), which contains 13 items and was designed to measure individuals’ malevolent creativity through the behavior of daily lives. A total of 958 participants from different regions of China completed the MCBS in an online fashion. Cronbach’s α coefficient, using the 908 MCBSs with entirely complete data, indicated that the MCBS had satisfactory reliability. Exploratory factor analysis (EFA) and confirmatory factor analysis (CFA) revealed that the MCBS had 3 dimensions: hurting people, lying, and playing tricks. MCBS scores were positively correlated with individuals’ aggression, openness, extraversion, and scores on the Runco Ideational Behavior Scale (RIBS). MCBS scores also predicted individuals’ malevolent creativity performances when solving realistic, open-ended problems. The MCBS has a simple response medium and scoring procedure. This, along with the adequate psychometric properties uncovered here, indicates that it is a useful tool for research on malevolent creativity. Given that the MCBS contains a relatively small number of categories and items, further research could expand the categories of items and develop and test more items. Moreover, it would be useful to test MCBS’s reliability and validity with other criteria. Perhaps future research could obtain actual MC data from criminal or other unambiguously malevolent samples.

## Introduction

The traditional definition of creativity focuses on the originality and appropriateness of people’s creative products and the ability to generate novel and effective ideas (Sternberg and Lubart, 1996; Runco and Jaeger, 2012). More than 50 years ago, Stein (1953) pointed to the societal impact of creative products (also see Mumford and Gustafson, 1988); as well, Rogers (1954/1959) pointed out that creativity could have both positive and negative purposes. The negative and anti-social side of creative received very little attention until the 1990s, when both creativity in the moral domain (Gruber, 1993) and the dark side of creativity (McLaren, 1993) were introduced.

Creative behavior can only be understood as positive or negative when intentions are taken into account (Runco, 1993). This allows the moral and dark sides of creativity to be distinguished from one another. Also, it leads to a distinction between malevolent creativity (MC) and negative creativity (NC). The former involves the application of original ideas to purposely harm others, often to gain an unfair advantage through manipulation, threat, or harm (Cropley et al., 2008; Cropley, 2010; Harris et al., 2013). NC, on the other hand, refers to creativity that is harmful to others without malevolent intentions (James et al., 1999).

Research in this direction is on the rise, with investigations of creative crime, terrorism, and deception. The clearest examples of MC involve crime and terrorism (Eisenman, 2008; Cropley and Cropley, 2011). Yet MC is not exclusive to criminals and terrorists. Everyone may have potential MC ideas. It may be a part of day-to-day life, at least in the form of lying, betrayal, deception, playing tricks, and so on (James et al., 1999; Spooner, 2008; Gill et al., 2013; Harris and Reiter-Palmon, 2015). Previous research demonstrated that people tended to show more MC in unfair conditions (James et al., 1999). The personality traits of physical aggression and conscientiousness (Lee and Dow, 2011), implicit aggression (Harris and Reiter-Palmon, 2015), and emotional intelligence (James et al., 1999) were also related to MC.

Three kinds of methods have been used to assess MC. James et al. (1999) asked participants to propose novel solutions to problems that suggest malevolence (e.g., “try to propose novel solutions as many as possible to slander competitor companies in front of your potential client”). The number of ideas was used as an indicator of people’s MC. Participants’ ideas were regarded as malevolent by default because they were set to be solutions of doing malevolent things. The problem with this approach is that the ideas generated for malevolent problems may be neutral or even benevolent (James et al., 1999). Thus, MC, defined such that it reflects both malevolence and creativity, was not perfectly measured with this method.

Lee and Dow (2011) used a second method to assess MC. It relied on the Alternative Uses Task (AUT; Guilford, 1967). Specifically, participants were asked to generate as many unusual or original uses as possible for common objects (e.g., brick). Then, the malevolent ideas (e.g., hitting others, or poison others after being grounded into powder) were selected by two raters (whose agreement was later determined). Afterward, a proportion score was calculated by the total number of all ideas divided by the number of malevolent ideas. This score was used as an indicator of MC. This method is similar to that used by Dudek and Verreault (1989), who were interested in the aggressive tendencies of individuals as they solved divergent thinking tasks. It is also consistent with the appropriateness scoring of divergent thinking used by Runco and Charles (1993) and Runco et al. (2005) —though of course malevolent ideas would be inappropriate rather than appropriate. In the research of Lee and Dow (2011), malevolent uses for common objects were not necessarily original. For example, hitting others with a brick is a malevolent idea but not a novel idea. Consequently, such a method did not appropriately measure the originality when people generated malevolent ideas.

Harris et al. (2013) improved on the method used by Lee and Dow (2011) by asking trained raters to look for two dimensions (i.e., malevolence and originality). Raters used a 6-point scale with 0 to 5 representing “not malevolent/original at all” to “malevolent/original very much.” Ideas scored more than or equal to 3 in both malevolent and original dimensions were counted as MC ideas. Ideas scored less than 3 on both dimensions, or scored more than or equal to 3 only in one of the dimensions, were not be counted as MC ideas. Although this method recognizes both malevolence and originality, as is vital for an accurate index of MC, it has its own limitations. One is that the MC ideas being selected based on such a criterion are quite few and may be none in one experiment trial; another is that this scoring procedure was sophisticated and required much time and efforts (Harris et al., 2013).

Notably, there is a limitation that applies to all three of the methods just described for the measurement of MC. Individuals may very well be reluctant to express creatively malevolent ideas. This is essentially the commonly-recognized tendency toward social desirable responding and action. In fact, creative ideas may break traditional rules, and creatively malevolent ideas may be doubly unacceptable to society (Mueller et al., 2012). If this is the case, individuals may hesitate admitting to their MC, even if they have malevolently creative ideas. They may conceal some or all of their ideas, or at least those that are in fact indicative of MC.

The three previous methods for the measurement of MC have their respective problems. This suggested the need for a new tool of measuring MC. It should have ecological validity, cover various forms of MC (e.g., deception, tricks, lies), and be easy to administer. Ideally it would minimize the tendency toward socially desirable responding; but that tendency actually plagues all self-reports, not just those which measure any expression of malevolence. Note here that there are reliable measures of lying used in the research (DePaulo and Kashy, 1998; DePaulo et al., 2004), even though those would have at least as much of a tendency toward socially desirable responding. Still, as is the case with the Runco Ideational Behavior Scale (RIBS; Runco et al., 2001, 2014), the measure developed in the present research focuses on day-to-day behavior, and some of the questions focus on behavior which are not always evil or illegal (e.g., playing tricks).

The new measure is the Malevolent Creative Behavior Scale (MCBS). Data are reported below to determine if the MCBS has good reliability, structural validity, and concurrent construct validity. Given the importance of creativity for society, and given people’s increasing interest in MC and the dark side of creativity, a new measure could prove to be enormously useful.

Several other measures were administered to check the concurrent construct validity of the MCBS. First, participants were asked to complete the RIBS. Since MC could be categorized as a particular kind of creative potential, the MCBS score was expected to show a significant but only moderate correlation with the RIBS score. Second, participants’ aggressive traits were measured by the Buss-Perry Aggression Questionnaire (BPAQ; Buss and Perry, 1992). It was revealed that aggressive individuals thought and solved problems in malevolently-biased ways (Anderson and Bushman, 2002; Lee and Dow, 2011); as well they exhibited high flexibility of generating malevolent idea as a form of defense (Harris and Reiter-Palmon, 2015).Therefore, the MCBS score was expected to be related with the BPAQ score. Third, it suggested that open people tend to be imaginative and curious so they are more creative (Feist, 2010). Empirical evidence showed that openness was positively related to creativity (Burch et al., 2006; Charyton and Snelbecker, 2007; Prabhu et al., 2008). Moreover, extraversion was demonstrated to benefit creativity, perhaps because extraverted individuals displayed a lower cortical arousal (Fink and Neubauer, 2008) or a higher dopaminergic activity (Stafford et al., 2010), which help to generate original ideas. Thus, participants were asked to complete the openness and extroversion subscales of NEO-PI-R. The openness and extroversion scores were expected to show significant correlations with the MCBS score. Fourth, some of the participants were required to complete MC performance tasks, the prediction being that the MCBS score would be significantly related to actual MC task performance.

## Materials and Methods

### Participants

A sample of 958 participants completed the MCBS, RIBS, AQ, and openness and extraversion sub-scales of NEO-PI-R. These data were collected online. The data of 50 participants had to be excluded from further analyses because of missing information. Thus, the final sample consisted of 908 participants (301 males, Mean age = 22.91, *SD* = 4.27). The participants attended this study voluntarily without compensation. To determine if the MCBS score could predict individuals’ MC performance, another other 128 participants (26 males, Mean age = 20.21, *SD* = 1.11) were recruited to complete the MCBS and solve a MC problem in the lab. These participants received approximately 3 US dollars for their participation (after the experiment). All of participants were native Chinese speakers. The protocol of the experiment was approved by the Institutional Ethics Committee at East China Normal University.

### Measurements

All questionnaires were released on the China Sojump Website (a public online platform to release questionnaires). Participants can either complete the questionnaires on computers or mobile phones. Participants in this study reached the questionnaires through the website or the We Chat APP. All data were stored in and downloaded from the website database. In order to ensure data authenticity, detailed introductions were conducted to emphasize participants that they should take the study seriously and that all of and would not be individually interpreted.

#### The Malevolent Creativity Behavior Scale (MCBS)

The MCBS was developed by first carefully examining the relevant literature (e.g., James et al., 1999; Spooner, 2008; Lee and Dow, 2011; Gill et al., 2013; Harris and Reiter-Palmon, 2015). Experts on MC were also consulted for ideas about indicative behaviors. This led to 20 MC behaviors that could occur in day-to-day life (e.g., deceptions, tricks, lies, betray, revenge, rumor mongering, etc.) Items were then composed to capture each of these behaviors in a format that was consistent with survey research. Afterward, two experts on MC and two non-experts were required to discuss the 20 items to eliminate redundancies. They suggested that seven items were superfluous and should be deleted. Thus, there were 13 unique and broadly understood items left. These 13 items were organized in the MCBS, each with a 5-point Likert. Respondents are asked to choose one number according to the frequency of each item in their own daily lives (0 = never, 1 = few times, 2 = sometimes, 3 = often, and 4 = usually). The sum score of all 13 items is referred to be the MCBS score; the higher, the greater MC.

#### The Runco Ideational Behavior Scale (RIBS)

The RIBS focuses on ideation that may occur in daily life (e.g., “how often do you have ideas for rearranging the furniture in your home?”). The short form of the RIBS was adopted in this study, which contains 19 items and uses 5-point Likert type scale (0–4, ranging from “never” to “just about every day”). Participants were asked to choose one number according to the frequency of each item in daily life. The sum score of 19 items is the creativity ideation score. Runco et al. (in press) reported an inter-item reliability of 0.90 for the RIBS, which is comparable to the reliabilities from other samples (e.g., Chand O’Neal et al., 2015; Paek et al., in press). The reliability of RIBS in the present investigation was entirely satisfactory (Cronbach’s α = 0.88).

#### The Buss-Perry Aggression Questionnaire (BPAQ)

The BPAQ (Buss and Perry, 1992) contains 29 items and uses 5-point Likert type scale, from 1 to 5 (“not at all” to “very much”). The Chinese revised BPAQ (Lv et al., 2013) contains 22 items, which has satisfactory internal consistency reliability (Cronbach’s α = 0.89), test-retest reliability (r = 0.91), and structural validity. The internal consistency reliability of BPAQ (α = 0.72) was satisfactory in this study.

#### The Openness and Extroversion Subscales of NEO-PI-R

The openness and extroversion subscales each contain 48 items (Costa and McCrae, 1992), with 5-point Likert type scale from 0 to 4, ranging from “not at all” to “very much”. The Chinese revised NEO-PI-R (Dai et al., 2004) shows that both openness and extroversion subscales have satisfactory internal consistency reliability (α >0.77), test-retest reliability (r >0.80), and structural validity. In the current study, the internal consistency reliability of openness subscale (α = 0.86) and extroversion subscale (α = 0.86) were also satisfactory.

#### The Malevolent Creativity Task

Because participants in this study were college students, a MC problem was written such that it could easily occur in the context of a college campus. That is, “Ming (a boy’s name) walked on his way one day. Wei (a boy’s name) was running in a hurry and bumped into Ming, and Ming’s computer dropped on the ground and broke. Wei criticized Ming and ran off without saying that he was sorry, which made Ming very angry.” Participants were asked to “propose as many ideas as possible to help Ming revenge himself on Wei without being discovered.” As the method adopted in the previous study (James et al., 1999), we calculated the number of solutions that participants generated for this problem, which was used as the indicator of participants’ MC.

### Statistics Analyses

The sample of 908 participants was divided into two equal parts, each of which contains 454 sets of data for EFA and CFA.The EFA and CFA were conducted using SPSS 17.0 and AMOS 21.0, respectively. The Kaiser-Meyer-Olkin (KMO) measure of sampling adequacy and Bartlett’s test were conducted to confirm that the data were appropriate for EFA and CFA. The reliability of all questionnaires was tested through Cronbach’s α coefficients (and was reported above). In addition, correlation analyses were conducted to test whether the MCBS scores were related to the RIBS, AQ, openness, extroversion, and the MC task performance. A regression analysis was conducted to explore whether these variables could predict the MCBS scores.

## Results

### Exploratory Factor Analysis (EFA)

The KMO measure of sampling adequacy was 0.87, indicating that the sample data were appropriate for factor analysis. Bartlett’s test (Approximately χ^2^ = 1792.23, df = 78, *p* < 0.01) also confirmed that the correlation matrix was not an identity matrix. Items that load exactly or greater than 0.40 in absolute value on relevant factors were retained. All of the 13 items were kept. The rotated component matrix of MCBS (see **Table 1**) suggested three factors in the EFA. These three factors accounted for 55.88% variance in the MCBS scores.

**Table 1:** Rotated component matrix of the Malevolent Creativity Behavior Scale (MCBS).

Item	Factor 1	Factor 2	Factor 3
Item 11	0.70		
Item 10	0.68		
Item 9	0.68		
Item 13	0.64		
Item 12	0.62		
Item 8	0.60		
Item 5		0.74	
Item 4		0.70	
Item 6		0.66	
Item 7		0.65	
Item 1			0.75
Item 3			0.69
Item 2			0.60


### Confirmatory Factor Analysis (CFA)

**FIGURE 1:**
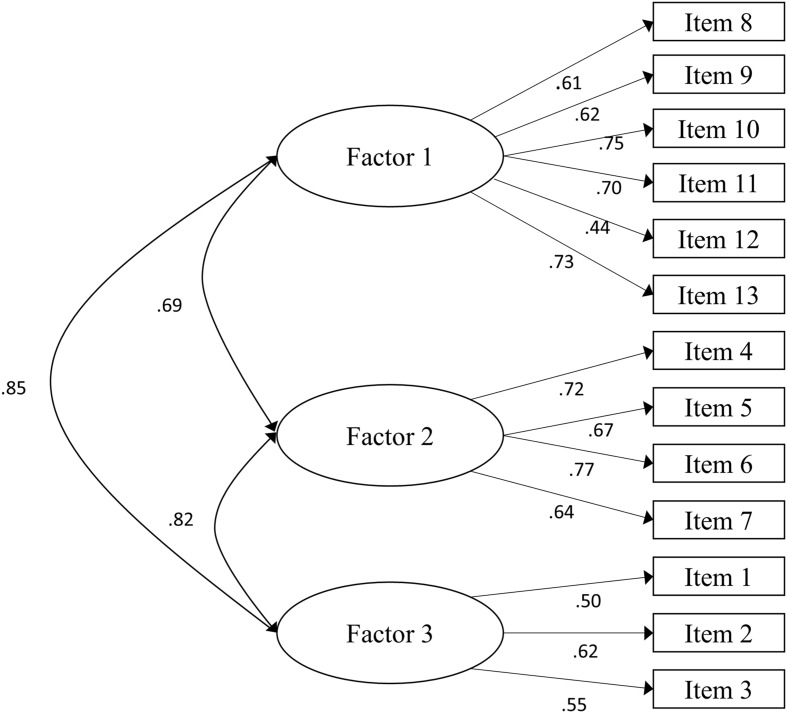
**The path diagram of standardized estimates of the Malevolent Creativity Behavior Scale (MCBS)**.

A CFA was conducted to test whether the data sample fitted the three-factor model uncovered by the EFA. Following former studies (Hu and Bentler, 2000; Schmitt, 2011; Yang and Montgomery, 2011), multiple indices were selected to evaluate model fitness, such as the chi-square statistics divided by the degrees of freedom (χ^2^/df), goodness of fit index (GFI), adjusted goodness of fit index (AGFI), root square means error of approximation (RSMEA), comparative fit index (CFI), Tucker-Lewis index (TLI), incremental fit index (IFI), and normed fit index (NFI). As shown in **Table 2**, each of these indices confirmed that the hypothesized model fit well with χ^2^/df = 2.86 < 3, RMSEA = 0.06 < 0.08, GFI = 0.95 > 0.90, AGFI = 0.92 > 0.90, NFI = 0.91 > 0.90, CFI = 0.94 > 0.90, IFI = 0.94 > 0.90, and TLI = 0.92 > 0.90. The path diagram of standardized estimates was illustrated in **Figure 1**.

**Table 2:** Model fitness indices of the Malevolent Creativity Behavior Scale (MCBS).

Indices	χ^2^/df	GFI	AGFI	RMSEA	NFI	IFI	TLI	CFI
	2.86	0.95	0.92	0.06	0.91	0.94	0.92	0.94


### Three Dimensions of the MCBS

As shown in **Table 3**, three factors of the MCBS were labeled “hurting people” (factor 1), “lying” (factor 2), and “playing tricks” (factor 3), according to the items they contained. “Hurting people” accounted for 22.58% variance in MCBS. “Lying” accounted for 18.83% of the variance in MCBS. And, “playing tricks” accounted for 15.1% of the variance in MCBS.

**Table 3:** The dimensions and items of the Malevolent Creativity Behavior Scale (MCBS).

Dimension	Item
Hurting people	(1) How often do you think about ideas to take revenge when being unfairly treated?
	(2) How often do you have ideas about new ways to punish people?
	(3) How often do you have ideas about how to suppress people who are in your way?
	(4) How often do you engage in an original form of sabotage?
	(5) How often do you have ideas to hurt yourself?
	(6) How often do you think about the strategies of hurting others in the rough world?
Lying	(7) How often do you fabricate lies to simplify a problem situation?
	(8) How often do you think about excuses to justify your wrongdoings?
	(9) How often do you tell lies without worrying about being nailed?
	(10) How often do you think of ways to conceal your misdoings from others?
Playing tricks	(11) How often do you have ideas about how to pull pranks on others?
	(12) How often do you play tricks on people as revenge?
	(13) How often do you think of ideas on the margins of rules, when conventional ways do not work?


### The Reliability of the MCBS

The Cronbach’s α coefficient for MCBS as a whole was 0.80, indicating its good reliability. The Cronbach’s α coefficients was also conducted to the three dimensions of MCBS. It was found that the reliability of three dimensions was acceptable (α = 0.80 for factor 1, α = 0.76 for factor 2; α = 0.61 for factor 3).

### The Predictive Validity of the MCBS

The regression analysis revealed that the MCBS score was significantly and positively correlated with participants’ performances on the MC task (*R* = 0.27, 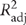 = 0.07, *F* = 10.17, *p* < 0.05; β = 0.27, *p* < 0.01). As shown in **Table 4**, the MCBS score was positively correlated to the aggression, openness, extraversion, and RIBS scores (*p*s < 0.01, 0.01, 0.05, and 0.01, respectively). The regression with the MCBS score as the dependent variable and the four variables above as the predictors (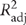 = 0.29; *F* = 84.12, *p* < 0.01) revealed that Aggression was a significant positive predictor (β = 0.50, *p* < 0.01), as was the RIBS sore (β = 0.22, *p* < 0.01).

**Table 4:** Correlations between the scores of MCBS, aggression, openness, extraversion, and RIBS.

	MCBS	Aggression	Openness	Extroversion	RIBS
MCBS	1				
Aggression	0.50^∗∗^	1			
Openness	0.09^∗∗^	0.27^∗∗^	1		
Extroversion	0.07^∗^	0.22^∗∗^	0.78^∗∗^	1	
RIBS	0.24^∗∗^	0.13^∗∗^	0.33^∗∗^	0.34^∗∗^	1


*^∗^*p* < 0.05, ^∗∗^*p* < 0.01.*

## Discussion

The present study developed the MCBS, a self-report scale of MC ideation, to measure MC in daily life. The MCBS contains 13 items, and was proved to have satisfactory reliability, structural validity, and predictive validity. It was demonstrated that individuals with higher MCBS scores did tend to think about malevolent and creative ways of solving problems. Compared to previous methods of measuring MC, the MCBS developed in this study is easy to be administered for individuals and groups because of its simple response medium and scoring procedure. As a result, the MCBS makes a significant contribution to the research on MC.

The EFA and CRA revealed that the MCBS had three dimensions: hurting people, lying, and playing tricks. “Hurting people” accounted for more than 20 percent variance in MCBS. This is consistent with the typical definition that MC is the application of original ideas to purposely harm others, often to gain an unfair advantage through manipulation, threat, or harm (Cropley et al., 2008; Cropley, 2010; Harris et al., 2013). Notably, the six items of “hurting people” involved in hurting self and others; this is in line with the previous finding that the objections of MC might be self or other stuffs (Harris and Reiter-Palmon, 2015).

“Lying” is also an important example of MC in daily life. On the one hand, previous studies revealed that individuals with higher creativity tend to be more dishonest (Walczyk et al., 2008; Silvia et al., 2011). Lying is also a problem solving strategy in some social situations; it requires flexibility and divergent thinking (DePaulo and Kashy, 1998; DePaulo et al., 2004). In this light, lying can be regarded as an activity embodied with creativity. On the other hand, lying is unacceptable in almost all cultures. It is unethical and may hurt others (Walczyk et al., 2008). Therefore, lying can be an indicator for MC in daily life.

“Playing tricks” can be creative as well. They require the effective surprise of creative thinking and are usually updated very frequently, given that once a kind of tricks has been discovered, it would be subsequently ineffective. The individuals must invent new tricks (Bailin, 1987). Playing tricks maybe not harmful as the other two dimensions of MC (lying and hurting people), but they bother others and are selfish. Playing tricks is an indicator for MC in daily life.

The MCBS score was positively related to the RIBS score (see **Table 4**). Since the RIBS was used to measure general creative ideation in daily life (not MC in particular), the significantly moderate (*r* = 0.24) correlation between RIBS and MCBS suggested that the MCBS was efficient in evaluating the “creativity” dimension of MC. Furthermore, the MCBS score predicted individuals’ performance on MC task. Participants with higher MCBS scores generated much more malevolent ideas in solving the problem. This result indicated that the MCBS was also effective in measuring the “malevolence” dimension of MC. In brief, the MCBS had satisfactory predictive validity in this study.

In addition, the MCBS sore was significantly correlated with the aggression score (see **Table 4**). Previous studies revealed that people with higher physical aggression produced more malevolent creative ideas in solving AUT problems than those with lower aggression, perhaps because aggressive people tend process open–ended tasks in a relatively malevolent perspective (Lee and Dow, 2011). Moreover, people who have high implicit aggression exhibit more MC than those with low implicit aggression (Harris and Reiter-Palmon, 2015). Possible explanations may include, on the one hand, an aggressive individual’s flexibility and use of MC ideas or solutions as a form of defense. On the other hand, implicitly aggressive people may be indifferent to some possible negative consequences and may possess unconscious mind sets to justify their behavior (Harris and Reiter-Palmon, 2015). The relation between aggression and MCBS observed in the current study suggests that the latter can be used to estimate the “malevolence” dimension of MC.

There are three limitations of this study. First, there were more female participants than males; the current results were mostly based on female participants. Further research should balance males and females in the sample, and also try analyzing gender differences in MC performance. Second, the dimension of “playing tricks” showed only moderate reliability (α = 0.61). This may because it contained a relatively small number of items (i.e., 3 items). Further research could expand this category of items and develop and test more items. Third, although the MCBS showed satisfactory reliability and validity, it still needs to be testified in research using other criteria. Further research could recruit criminals as participants, who were sentenced to prison for terrorism, mayhem, cheat, theft and so on. Perhaps actual MC data could be obtained in these criminal or other unambiguously malevolent samples. The present results are promising, but additional research would be informative.

## Author Contributions

NH provided the idea, designed this study and wrote the manuscript. MT and JY contributed to research design and data analysis. QW contributed to data collection. MR contributed to paper writing.

## Conflict of Interest Statement

The authors declare that the research was conducted in the absence of any commercial or financial relationships that could be construed as a potential conflict of interest.
